# Crystal Structure of Circular Permuted *Ro*CBM21 (CP90): Dimerisation and Proximity of Binding Sites

**DOI:** 10.1371/journal.pone.0050488

**Published:** 2012-11-30

**Authors:** Preyesh Stephen, Kuo-Chang Cheng, Ping-Chiang Lyu

**Affiliations:** 1 Institute of Bioinformatics and Structural Biology, National Tsing Hua University, Hsinchu, Taiwan; 2 Department of Medical Sciences, National Tsing Hua University, Hsinchu, Taiwan; 3 Graduate Institute of Molecular Systems Biomedicine, China Medical University, Taichung, Taiwan; University of South Florida College of Medicine, United States of America

## Abstract

Glucoamylases, containing starch-binding domains (SBD), have a wide range of scientific and industrial applications. Random mutagenesis and DNA shuffling of the gene encoding a starch-binding domain have resulted in only minor improvements in the affinities of the corresponding protein to their ligands, whereas circular permutation of the *Ro*CBM21 substantially improved its binding affinity and selectivity towards longer-chain carbohydrates. For the study reported herein, we used a standard soluble ligand (amylose EX-I) to characterize the functional and structural aspects of circularly permuted *Ro*CBM21 (CP90). Site-directed mutagenesis and the analysis of crystal structure reveal the dimerisation and an altered binding path, which may be responsible for improved affinity and altered selectivity of this newly created starch-binding domain. The functional and structural characterization of CP90 suggests that it has significant potential in industrial applications.

## Introduction

Glucoamylases (GA: EC 3.2.1.3, 1,4-α-D-glucan glucohydrolase) are amylolytic enzymes [1], possessing specific raw starch-binding ability through their carbohydrate-binding module (CBMs). Carbohydrate-binding modules (CBMs) are linked to GAs by an *O*-glycosylated linker sequence and enhance the amylolytic process by transporting the GAs to the surface of insoluble polysaccharides. GAs from *Rhizopus oryzae* (*RoGA*) posses an N-terminal starch binding domain (SBD) [2], [3], which belongs to CBM family 21 (*Ro*CBM21) [4], [5] and shares relatively low level similarity with the SBDs derived from other starch-processing enzymes. The CBMs, in general, tend to contain a characteristic feature termed the β-sandwich fold [6] with one or, more frequently, two distinct binding sites that exhibit site-dependent modes of carbohydrate binding [7]–[12]. Atomic force microscopy and site specific mutation of SBD in *Aspergillus niger* have shown that both binding sites are needed to induce a gross conformational change in amylose molecules [13]. *Ro*CBM21 has been proposed to have a cooperative mode of interaction to sugar molecules, which are dominated by hydrophobic-binding region created by aromatic residues namely Trp^47^, Tyr^83^, and Tyr^94^ at site I and Tyr^32^ and Phe^58^ at site II [10], [12]. Besides the hydrophobic interactions, numerous hydrophilic interactions and a continuous polysaccharide binding path connecting site I and site II has also been postulated [12].

Starch is the primary energy source in many plants and an important raw material for food and industrial applications. Starch consists 20–30% of amylose composed of α-(1→4) bound glucose molecules [14] and 70–80% of amylopectin composed of α-(1→4) glucan chains, that are connected by α-(1→6) linkages [15]. Both amylose and amylopectin can fold into helical structures [16], [17]. Amylose is more flexible in solution, which allows it to adopt a locally helical shape [18] with the structural features denoted as A, B, and V [19]–[21].

GAs possessing a starch-binding domain are responsible for ∼10% of starch hydrolysis taking place in nature [22]. SBDs retain their starch binding abilities even when separated from the catalytic domain [23], [24]. SBDs have important industrial applications such as in food processing [25], starch liquefaction [26], selective removal of starch from textiles [27], affinity purification of recombinant proteins [28]–[30], and improving efficiencies of non-amylolytic enzymes [31].

The specificities and sterioselectivities of amylolytic systems (GA-SBD complexes) determine the specific end products, eg., glucose and fructose [32]. An efficient and selective attack by the GA on the crude mixture of starch molecule is a rate-limiting step in the aforementioned applications. Protein-engineering of SBDs [33] and amylase [34], [35] to obtain candidates that can efficiently and selectively attack specific form of carbohydrates may find useful applications in starch processing industries.

We previously created a circularly permuted form of *Ro*CBM21 in which the original N and C termini of *Ro*CBM21 were joined and new termini were created at positions 89 and 90. This mutant, whose N- terminus starts with G90 of *Ro*CBM21, was named CP90 [33]. Unlike most other constructed circularly permutated proteins [36]–[39], CP90 had significantly enhanced binding affinity and improved selectivity toward long-chain carbohydrates (i.e., its affinity for β-cyclodextrin is less than that for amylose EX-I, which is less than that for soluble and insoluble starches) [33]. For the study reported herein, examination of the CP90 crystal structure, its biophysical properties, and its binding affinity for amylose EX-I has suggested a possible, alternative binding path that causes its altered binding behavior.

## Results and Discussion

### Characterization of CP90

Protein structure can be considered as an ensemble of many fluctuating micro-states [40]. The characteristic native structures of proteins are lost upon denaturation, which affects such general protein properties as solubility and specific properties such as those related to function [41]. A comparison of the CD spectra of *Ro*CBM21 and CP90 at 25°C indicates that *Ro*CBM21 contains a larger amount of secondary structure between pH 5.0 and 6.0 than does CP90, whereas the CP90 contains a larger amount of secondary structure between pH 4.0 and 5.0 (Figure S1). Guanidine-HCl denaturation is used to assess specific contributions of hydrophobic and nonionic interactions that contribute to a protein’s stability by comparison of the denaturation profiles of a native and corresponding mutated protein(s) [42]. Gdn.HCl denaturation (at pH 5.5) of *Ro*CBM21 and CP90 were monitored by using CD spectroscopy, observing the change in mean residue elipticities at 215 nm. At 3.8 M and 2 M guanidine-HCl, 50% of the *Ro*CBM21 and CP90 molecules, respectively, had been denatured (Figure S2). A summary of physical properties exhibited by *Ro*CBM21 and CP90 are shown in Table S1.

### Crystal Structure of CP90; The Dimer Interface

**Figure 1:**
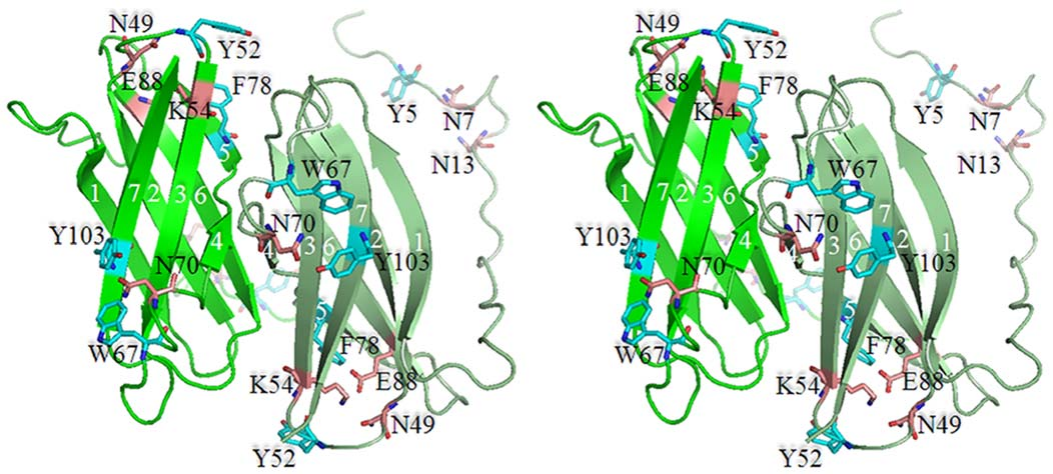
The Crystal Structure of CP90. A. A stereo view of CP90 crystal structure is shown. The ribbon diagram of chain A and B are colored green and pale green, respectively. The seven β-strands in each subunit are labeled 1 to 7 and the corresponding *Ro*CBM21 residues that bind β-cyclodextrin [12] are labeled (CP90 residue number are shown). The direct binding aromatic and hydrophilic residues are colored cyan and salmon, respectively. Y^5^, N^7^ and N^13^ (CP90 residue number are shown), which are corresponding binding residues in *Ro*CBM21 are placed in the N-terminal unstructured region of CP90.

**Figure 2:**
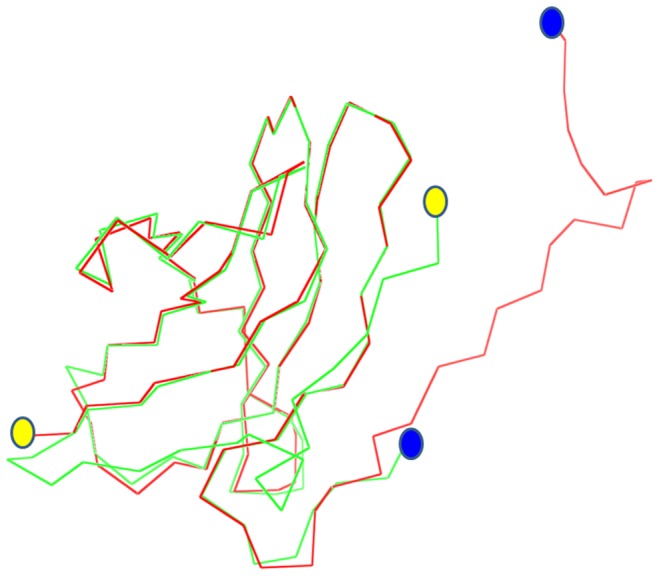
Structural superposition of CP90 (green) and *Ro*CBM21 (PDB code: 2VQ4, red). The blue and yellow circles represent the N-and C-termini, respectively, in the two proteins.

The first three dimensional (3D) apo*Ro*CBM21 [11] and β-cyclodextrin/G7- *Ro*CBM21 [12] structure have been resolved by NMR and x-ray crystallography, respectively. For this report, we collected diffraction data for CP90 to 1.86 Å resolution and solved its structure (PDB ID: 4EIB) by molecular replacement with the *Ro*CBM21 crystal structure (PDB ID: 2VQ4) as the search model. The data collection and refinement statistics are provided in Table 1. Only the φ-φ angles of Asp^13^ are found in the disallowed region of the Ramachandran plot (Figure S3), which belongs to the unstructured region (residues 1–28) in the N-terminal end of CP90. Parenthetically, we attempted to, but could not, express a truncated form of CP90 that lacked the N-terminal (1–28) residues, which meant that we could not explore any possible functional/structural role(s) involving these residues. The asymmetric unit of CP90 reveals a homodimer in which each unit is arranged in a slightly tilted antiparallel fashion (Figure 1). The oligomeric state of CP90 was further confirmed using analytical ultracentrifugation (see below). The *Ro*CBM21 and the CP90 share a similar overall topology. The superposition of CP90 crystal structure over the *Ro*CBM21 (Figure 2) shows that 88 residues aligned with 83% identity with a root mean square deviation (RMSD) of 0.702. CP90 crystal structure showed only 7 β-sheets while *Ro*CBM21 has 8 β-sheets. CD spectra (Figure S1) also reflected that CP90 contains quantitatively less secondary structure content of the *Ro*CBM21 under the solution conditions (pH 5.5) used to crystallize the CP90. The 7 β-strands of CP90 are arranged in an anti-parallel fashion within a monomer and between the monomers.

**Table 1:** Data Collection and Refinement Statistics.

*Category*		*Data Set*
***Data collection***	Wavelength(Å)	0.97622
	Temperature(K)	100
	Resolution(Å)	30–1.86 (1.89–1.86)^a^
	Space group	P4
	Unit cell dimensions	
	a, b, c(Å)	66.94, 66.94, 44.36
	α, β, γ(°)	90, 90, 90
	No. of unique reflections	16656
	No. of observations	124560
	Redundancy	7.5
	Completeness (%)	99.9
	Mean I/σI	49.4
	R _merge_ b	0.034
***Refinement***	Resolution range (Å)	66.94– 1.82
	Number of reflections	14960
	R _work_ c	0.169
	R _free_ c	0.241
	Average B-factor (Å^2^)	18.911
	R.m.s.d bond lengths (Å)	0.013
	R.m.s.d bond angles (°)	1.381
***Ramachandran Plot (%)***	Residues in most favored region	83.5
	Residues in the additional allowed region	14.9
	Residues in generously allowed region	0.5
	Residues in disallowed region	1

a Values in parentheses are for the highest-resolution shell.

b *R_merge_* = Σ_hkl_Σ_i_ |*I*_i_ (hkl)−<*I*(hkl)>|/Σ_hkl_Σ_i_ *I*_i_ (hkl).

c *R_work_* and *R_free_* = Σ||F_obs_|− |F_calc_||/Σ|F_obs_|,where *R_free_* was calculated over 5% of amplitudes.

that were chosen at random and not used in refinement.

d Ramachandran analysis from PROCHECK [47].

**Figure 3:**
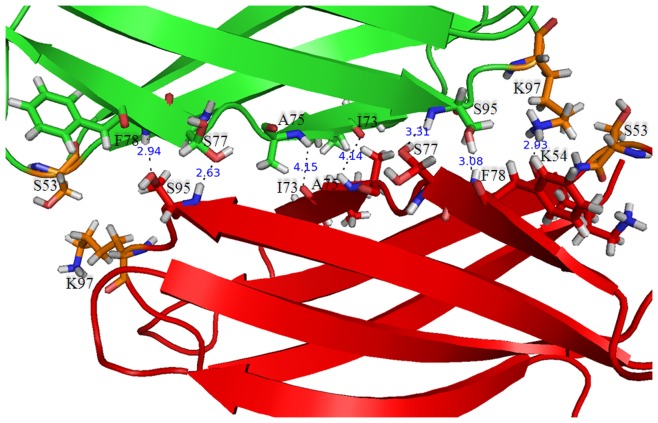
A ribbon diagram of CP90 showing the dimer interface. Subunit A and B are colored green and red, respectively. Amino acid residues in the dimeric interface are shown as sticks models. The hydrophobic interactions are colored orange. Hydrogen bonds are shown by dashed lines with the values for the distance separations for the interacting atoms shown.

To explore the various energy forces that stabilize dimeric CP90, we used Dimplot [43] and mapped the interactions across the dimer interface. There was no significant electrostatic interaction between the monomers. The major interface contacts involve hydrophobic and hydrogen bond interactions, which were mapped onto the CP90 structure using Pymol (Figure 3). The possible hydrophobic interactions at the interface are through the side chain methylene groups of Lys^97^ and Ser^53^. There are 7 hydrogen bonds formed at the dimer interface which are made by hydrophobic residues (Phe^78^, Ile^73^, Ala^75^), hydrophilic residues (Lys^54^, Lys^97^) and amino acids with polar neutral side chains (Ser^95^, Ser^77^). The dimer interface is completed by 18 hydrogen bonded water molecules buried within the cavity that fill the dimer interface (Table S2). The water mediated hydrogen bonds were observed at the Ser^39^O^γ^, Lys^54^N^ζ^, Val^56^NH, Thr^57^O^γ^, Ser^64^O^γ^, Asn^66^N^δ^, Asn^72^N^δ^, Ser^93^NH, Lys^97^NH, Lys^97^N^ζ^ of subunit A and Thr^40^O^γ^, Val^56^NH, Tyr^60^OH, Asn^66^N^δ^, Asn^68^N^δ^, Asn^72^N^δ^, Asn^72^NH, Lys^97^NH of subunit B.

### CP90; Dimer to Tetramer on Ligand Binding

**Figure 4:**
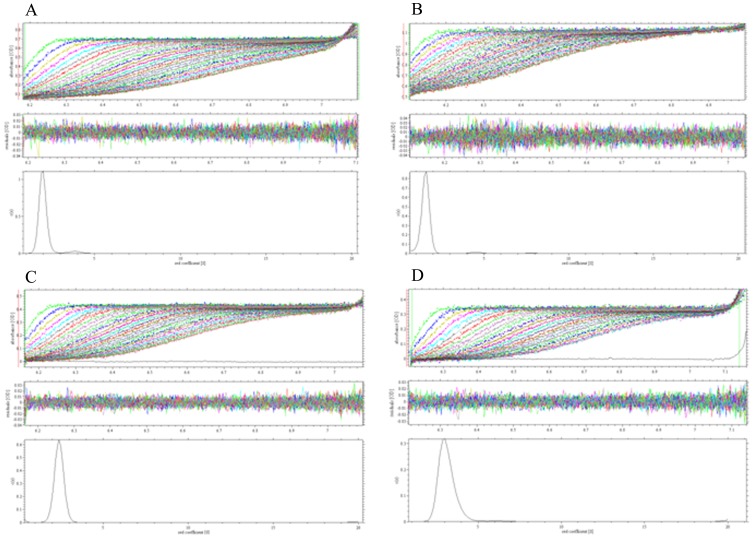
Sedimentation velocity analysis. **A,** *Ro*CBM21; **B,** *Ro*CBM21 & amylose EX-I; **C,** CP90; **D,** CP90 & amylose EX-I. The three panels in each experiment represent the trace of absorbance at 280 nm during the sedimentation, the residues of the model fitting, and the sedimentation coefficient distribution for all species.

To probe the oligomeric state of CP90 and the *Ro*CBM21, both the proteins were subjected to analytical ultracentrifugation in the presence and absence of amylose EX-I. As shown in Figure 4, as well as Table 2, *Ro*CBM21 is monomer and it became dimer in presence of amylose EX-I. In contrast, CP90, shown to be dimer, became tetramer in presence of amylose EX-I. The sedimentation coefficient for apo*Ro*CBM21 is 1.549 S and the molecular mass is 11 kDa (Figure 4A & Table 2), whereas in presence of amylose EX-I, the sedimentation coefficient is 1.988 S and the molecular mass is 21 kDa (Figure 4B & Table 2). The sedimentation coefficient for apoCP90 is 2.316 S and the molecular mass is 24 kDa (Figure 4C & Table 2), whereas in presence of amylose EX-I the sedimentation coefficient is 3.007 S and the molecular mass is 46 kDa (Figure 4D & Table 2). Unlike monomeric apo*Ro*CBM21, CP90 is, therefore, a dimer in its apo form and tetramerizes on ligand binding.

**Table 2:** Sedimentation velocity analysis.

	*Ro*CBM21	*Ro*CBM21 & amylose EX-I	CP90	CP90 & amylose EX-I
Sedimentation coefficient (_s20,w_) (S)	1.549	1.988	2.316	3.007
Molecular mass	11 kDa	21 kDa	24 kDa	46 kDa

The sedimentation coefficients (S) and molecular masses are listed for *Ro*CBM21 and CP90 in their apo and ligand-bound forms. The experiments were repeated thrice with similar results obtained using different batches of purified proteins. One representative set of the data is shown here.

The dimeric state of apoCP90 in solution as assessed by ultracentrifugation is entirely consistent to its crystal structure. The apparent driving forces for dimerization of CP90 have been delineated in detail (above). The dimerization of *Ro*CBM21 upon ligand binding is evident in the G7-*Ro*CBM21 complex crystal structure (PDB ID: 2V8M). The detailed analysis of this complex crystal structure is discussed elsewhere [12], as the binding of one maltoheptaose (G7) at the cooperative binding sites of *Ro*CBM21 can results in one *Ro*CBM21 to share each binding site with the symmetry-related molecule in the crystal such that two *Ro*CBM21 together hold one sugar ligand. Possibly the CP90 dimer tetramerizes by binding amylose EX-I intermolecularly in a manner similar to that of the *Ro*CBM21 protomer.

The oligomeric state of CP90 was assessed at pH 2.5, 3, 4, 5, 6, 7, 8, 9, and 10 and in the presence of 0-, 1-, 5-, and 10-fold excess of amylose EX-I. Under all pH conditions, apoCP90 was dimeric, whereas it was tetrameric in the presence of ligand at all the tested configurations (data not shown). It appears, therefore, that ligand binding drives dimerization of *Ro*CBM21 and tetramerization of CP90.

### Amylose EX-I Binding

**Figure 5:**
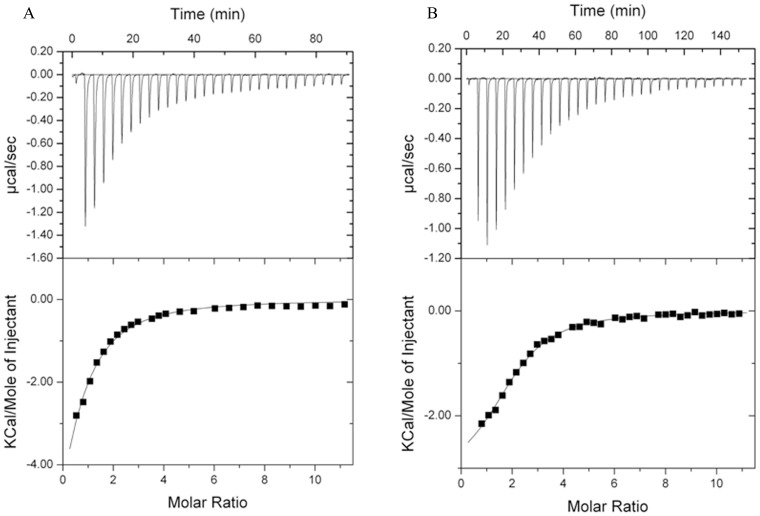
Isothermal titration calorimetry of *Ro*CBM21 and CP90 with amylose EX-I as the ligand. A. Titration of *Ro*CBM21 and **B.** CP90 with Amylose EX-I. The top half of each panel shows the raw titration data, and the lower half shows the integrated titration curves corrected for the heat of dilutions.

Amylose EX-I was used as the moderate molecular mass ligand to conveniently use in isothermal titration calorimetry (ITC) studies to determine the thermodynamic forces that drive the interactions of *Ro*CBM21 and CP90 with their ligands. As noted above, sites I and II of *Ro*CBM21 participate in a cooperative binding mode [12] and a model for the interaction between starch binding domain (SBD) and amylose that incorporates both binding sites as essential for the conformational change that occurs in starch hydrolysis, have been proposed [13]. Notably, therefore, for our ITC experiments, a two-step, fixed-binding-sequence equation, which is independent of the parameter N defining the number of binding sites, provided the best-fit to the titration data (Figure 5A&B). For both *Ro*CBM21 and CP90, the corresponding K_a1_ value is larger than that for *K*_a2_. Additionally, the *K*_a1_ and *K*_a2_ values for CP90-amylose EX-I binding are ∼2.5 times and ∼5 times greater than those of *Ro*CBM21 (Table 3). A moderate amount of heat was released when *Ro*CBM21 and CP90 bound with Amylose EX-I, indicating that the binding interactions had only moderate enthalpic contributions (−3.9**kcal/mol for both the Δ*H1 and* Δ*H2* of *Ro*CBM21 while CP90 had −2.5 kcal/mol and −3.9 kcal/mol for Δ*H1 and* Δ*H2*, respectively). In contrast, a highly favorable entropic contribution (Δ*S = *9.8 and 3.4 cal/mol/K for *Ro*CBM21 while CP90 had 16.4 and 6.6 cal/mol/K for Δ*S1 and* Δ*S2*, respectively) was observed, indicating the dominating hydrophobic interactions between the protein and carbohydrates, which previously been stated for *Ro*CBM21 [10], [12].

**Table 3:** Affinity of the RoCBM21 and CP90 for amylose EX-I as determined by isothermal titration calorimetry.

	*K_a_* *(10^5^ M^−1^)*	K_d_(µM)	ΔH(kcal/mol)	ΔS(cal/mol/K)	ΔG(kcal/mol)
***Ro*** **CBM21**					
**Site I**	1.240±0.079	8.064±0.051	−3.999±0.062	9.890	−6.947
**Site II**	0.045±0. 002	222.222±5.018	−3.947±0.140	3.490	−4.987
**CP90**					
**Site I**	2.730±0.404	3.663±0.055	−2.521±0.086	16.400	−7.411
**Site II**	0.234±0.015	42.735±0.275	−3.964±0.110	6.690	−5.958

Data are reported as the averages of three independent titrations.

### Ligand Binding Sites; Site-directed Mutagenesis

The super-positioning of *Ro*CBM21 and CP90 showed that substantial shift in the orientation of amino acids either at site I or site II had not occurred. However, two of the aromatic residues (Tyr^93^ and Tyr^94^) and one hydrophilic residue (Asn^101^), which form part of binding pocket in the *Ro*CBM21 have been moved to the N-terminal unstructured region in CP90 (see the unstructured region in Figure 1). Although the overall electrostatic potential is unchanged by circular permutation, a remarkable variation (positive to negative) near the binding site II (Figure S4) is observed. The dominant negative charge near the binding site II is caused by the replacement of positively charged Lys^91^ in *Ro*CBM21 to the N-terminal unstructured region of CP90 and the additional negative charge provided by the C-terminal carboxyl, which is proximal to the site II of CP90. The solvent accessibilities predicted using SABLE server [44] indicated that all corresponding binding site residues of *Ro*CBM21 in CP90 are also solvent accessible (Figure S5).

**Figure 6:**
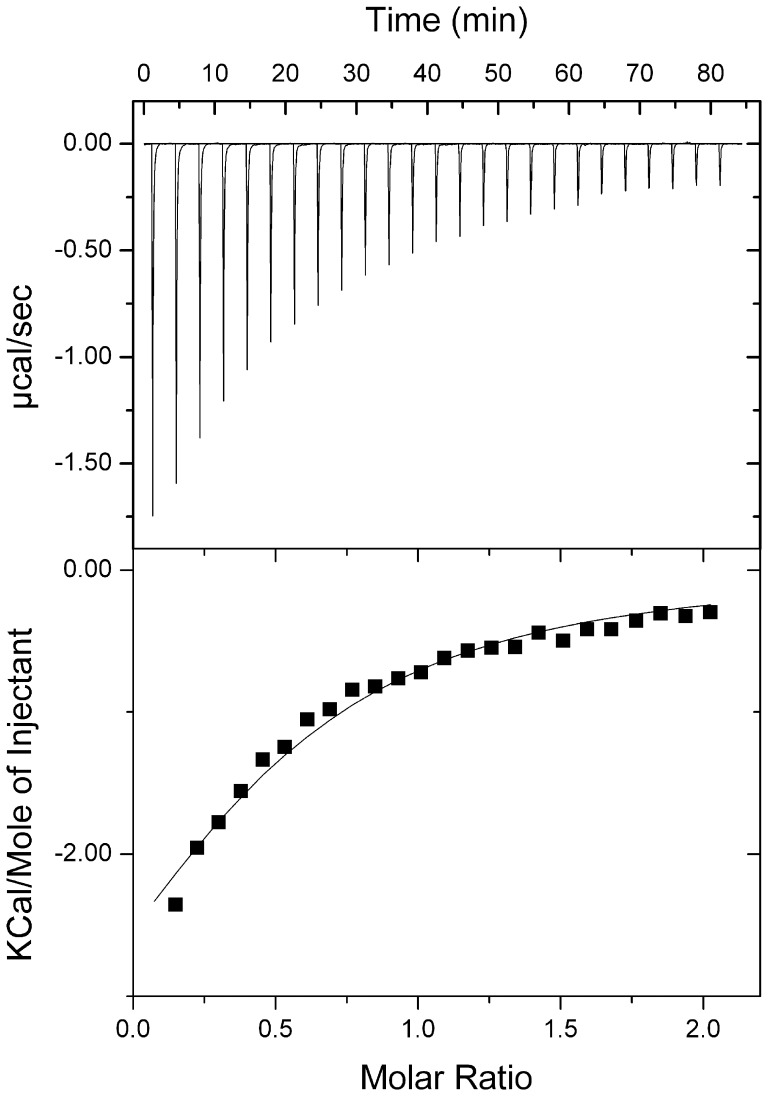
Isothermal titration calorimetry of Y52A of CP90 with Amylose EX-I. The top half of each panel shows the raw titration data, and the lower half shows the integrated titration curves corrected for the heat of dilutions.

Trp^47^ (at site I) and Tyr^32^ (at site II) in *Ro*CBM21 have been shown to be the major binding site residues involved in binding to starch and β-cyclodextrin [12]. A recent study performed to differentiate the relative importance of Trp^47^ and Tyr^32^ on the binding of soluble and insoluble carbohydrate molecules demonstrated that the Tyr^32^ is the major binding site residue, while Trp^47^ had only a weaker binding affinity to amylose EX-I [45]. Therefore, we created a CP90 mutant Y52A (this tyrosine corresponds to Tyr^32^ in *Ro*CBM21) to ensure that the major binding pocket is retained. The molecular mass of the mutant protein was determined by matrix-assisted laser desorption/ionization mass spectroscopy, which was in agreement with the theoretical molecular mass. The secondary structure content of Y52A verified by CD spectroscopy also was in agreement to the observed secondary structure of CP90 [33]. Using ITC, the binding curve for Y52A and amylose EX-I was best fit with a one-site binding equation (N = 0.5) and a *K*_d_ value of 294 µM, indicating that the binding site in CP90 that has the greater affinity for amylose EX-I was absent in Y52A (Figure 6 and Table 4). The binding affinity (294 µM) observed for site II mutant (Y52A) could have arisen from the corresponding site I residues (W^67^ in CP90 corresponds to W^47^ in *Ro*CBM21). Therefore, the CP90 has a similar binding mechanism of *Ro*CBM21 and the major site (site II) of *Ro*CBM21 is retained as the principal binding pocket.

**Table 4:** Affinity of the CP90 mutant Y52A for amylose EX-I as determined by isothermal titration calorimetry.

*K_a_* *(10^5^ M^−1^)*	K_d_(µM)	ΔH(kcal/mol)	ΔS(cal/mol/K)	ΔG(kcal/mol)
**CP90_Y52A**				
0.034±0.012	294.117±9.230	−4.952±0.168	−0.238	−4.891

Data are reported as the averages of three independent titrations.

### Proposed Alternate Binding Path; Polysaccharide Binding

**Figure 7:**
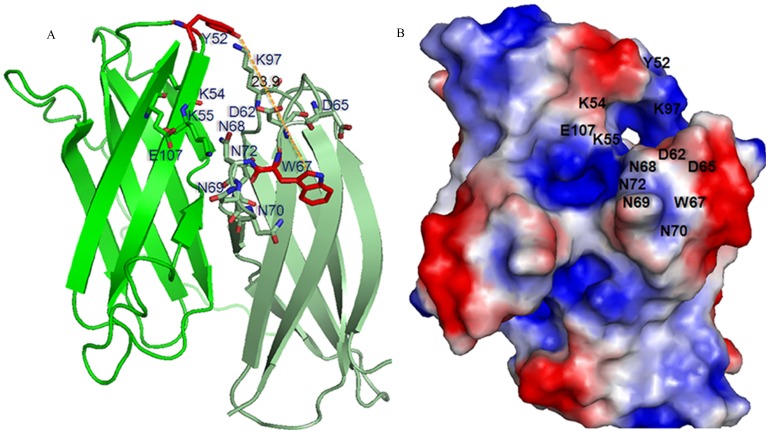
The alternate path for polysaccharides binding. **A.** A continuous polysaccharide-binding path mapped onto the CP90 ribbon diagram**.** Subunit A and B are colored green and pale green, respectively. The amino acids in the binding path are shown as stick models. The major aromatic binding residues Y^52^ and W^67^ are colored red. The distance (Å) between these aromatic residues are labeled and shown as a dotted yellow line. **B.** Electrostatic surface potential of the CP90 displayed using the program Pymol (DeLano Scientific; http:/pymol.sourcefirge.net/). The negative potentials colored red and positive potentials colored blue. The residues of binding path in the electrostatic surface are labeled.

Two continuous, surface binding paths involving Asn^50^, Tyr^83^, Tyr^94^, Tyr^93^ and Tyr^67^ (aromatic-dominated) or Asn^50^, Lys^85^, Glu^87^, Lys^35^, and Lys^34^ (hydrophilic) had been proposed between the binding sites (site I and site II) of *Ro*CBM21 [12]. These binding paths may not be important for soluble and small carbohydrates, such as β-cyclodextrin and G7, but are important for those of greater molecular mass carbohydrates, such as amylose EX-I [12], which can spread from site I to site II through these binding paths. Circular permutation had moved two of the aromatic-dominated binding path residues in *Ro*CBM21 (Tyr^93^ and Tyr^94^) to the N-terminal unstructured region. The Tyr^93^ and Tyr^94^ are placed at Tyr^4^ and Tyr^5^ in CP90. The loss of these major midpoint residues that might link the binding sites could force the CP90 to not adopt the aromatic-dominated binding path. Although CP90 can adopt the second binding path (hydrophilic binding path), the dimerisation provides an alternate binding path to connect the binding site I and II. The proposed alternate binding path for CP90 involves the hydrophilic residues Lys^54^, Lys^55^, Glu^107^ near the binding site residue Tyr^52^ in one subunit that connect to the next subunit through hydrophilic core Lys^97^, Asp^62^, Asp^65^, Asp^68^, Asn^69^, Asn^70^ and Asn^72^ situated near second binding site residue Trp^67^ (Figure 7A). From the surface electrostatic potential distribution of the CP90 (Figure 7B), a continuous hydrophilic surface is observed, which involves the residues mentioned in the proposed binding path. The length of this potential polysaccharide binding path is ∼25–30 Å, whereas the binding path for *Ro*CBM21 through hydrophilic residues was ∼30–35 Å and through the hydrophobic residues was ∼45–60 Å. Occam’s razor suggests to adopt a shortest binding path through which CP90 would accommodate one amylose EX-I at both binding sites. In a manner similar to that of *Ro*CBM21 [12], the binding of amylose EX-I at the cooperative binding sites would result in two CP90 dimer together hold the amylose EX-I such that to become the CP90 tetramer.

### Conclusions

The functional and structural characterization of CP90 substantiates it as a novel and potential starch binding module for industrial applications. The mutation study that replaced Tyr^52^ with an alanine in CP90 confirmed that the binding sites of the wild type protein are maintained in CP90. This paper also states apo*Ro*CBM21 is monomeric in solution and dimerizes in presence of amylose EX-I, whereas apoCP90 is a dimer and tetramerizes in presence of amylose EX-I. The detailed analysis of CP90 crystal structure revealed an alternate binding path for CP90-amylose EX-I binding, which could be why the binding affinity and selectivity of CP90 for longer-chain carbohydrates are enhanced in comparison with those for *Ro*CBM21. To our knowledge, CP90 is the first engineered starch binding module demonstrated to have an altered selectivity with an increased affinity towards starch. We hypothesize that the CP90 will find applications in most of the starch processing industries where SBDs are currently used. Our study also validates circular permutation as an engineering tool to improve the functional characteristics of other CBM families.

## Materials and Methods

### Cloning of Circular Permutated *Ro*CMB21 Genes

The nucleotide sequence of *Ro*CBM21 [10] was used for getting the clone of CP90 in pET28a at *Nco*I and *Xho*I [33]. The mutant Y52A was obtained by point mutation using complementary primers containing the desired mutations (Forward: 5- GTCAAGAACATTGCTGCCTCCAAGAAAGTTACT-3 and reverse: 5- AGTAACTTTCTTGGAGGCAGCAATGTTCTTGAC-3) and Pfu ultra DNA polymerase (Agilent). The DNA sequence of CP90 and Y52A were verified at Mission Biotech Co., Ltd. Taiwan. All the materials required for PCR (except primers) were purchased from MDBIO, Inc. (USA). Primers were from Mission Biotech (Taiwan).

### Protein Expression and Purification

*Escherichia coli* BL21 (DE3) cells (Novagen) were each transformed with expression vectors containing CP90/Y52A gene, then inoculated into Luria-Bertani medium containing 100 µg/ml ampicillin and cultured at 37°C until the OD_600_ of each culture reached 0.6. Protein expression was induced by the addition of 0.2 mM isopropyl β-d-thiogalactoside (final concentration), and the cultures were incubated at 18°C for an additional 16–18 h. Cells were harvested by centrifugation at 7000× *g*, 4°C for 10 min. Pellets were each suspended in 30 ml of 20 mM sodium acetate, pH 4.5, and homogenized (EmulsiFlex-C5). After centrifugation at 16,000× *g*, at 4°C for 30 min, the supernatants were each chromatographed through an *AKTA prime* FPLC/Hitrap SP column system (GE Healthcare, UK), which had been washed with sodium acetate, pH 4.5, and purified using sodium acetate, pH 4.5, containing <50 mM NaCl. The sodium acetate and the sodium chloride were purchased from USB Corporation (USA). The purified proteins were observed in 15% SDS PAGE and the molecular masss of the purified proteins were measured using an autoflex III smart beam MALDI-TOF (Bruker). Protein concentrations were assayed by a BCA (bicinchoninic acis) reagent kit (Pierce).

### Circular Dichroism (CD)

Circular dichroism spectra were acquired using an Aviv 202 spectropolarimeter (AVIV Biomedical, Lakewood, NJ) and a 1-mm path-length cuvette. The far-UV CD spectra of the proteins, each corrected for the contribution of the solvent, (30 µM protein, 20 mM sodium acetate, pH 5.5) are reported as mean-residue ellipticity ([θ], deg•cm^2^•dmol^–1^). Ellipticities were measured between 190 nm and 260 nm at 1-nm intervals. The pH titration was performed by incubating each protein sample (30 µM, in 20 mM sodium acetate) in various pH solutions from pH 3 to pH 11 at 25°C. The chemical-induced unfolding experiments were carried out by treating the protein sample at different concentrations of Gdn.HCl at 25°C. The unfolding curves were fitted to a nonlinear least-squares analysis using the equation [46].
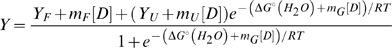
where *Y* is the value of the spectroscopic property of protein at a given Gdn.HCl concentration [D], *Y*_F_ and *Y*_U_ denote the intercepts, *m*_F_ and *m*_U_ are the slopes of the baselines of the native and unfolded states, respectively, *m*_G_ is a measure of the dependence of Δ*G*° on [D], and Δ*G*°(H2O) is the free energy change in the absence of denaturant.

### Crystallization and Structure Determination

Protein crystal- CP90 apo (12 mg/ml) was grown at 20°C in 1.26 M (NH_4_)_2_SO4, acetate pH 4.5 (0.1 M), NaCl(0.2 M) at a 1∶1 ratio of protein to mother liquid. Clear formation of single crystal formation was observed within 90 days of incubation and was flash frozen into liquid N_2_. The crystals were mounted on beamline BL13C1, NSRRC (Taiwan). Data were collected to 1.8 Å using ADSC Quantum-315 CCD Area Detector at a wavelength of 0.97622 Å. A total of 180 images were collected and were processed using HKL2000. The part (20–109 amino acids) of CP90 apo structure was solved by molecular replacement using the program Phaser. The crystal structure of CBM21 (PDB code: 2V8L) was used as the search model. The manual modification and extension of missing parts were carried out using *Coot* and refinement cycles were carried out using REFMAC5. Solvent water molecules were added using Coot and checked manually. The overall geometry of the final structure was assessed by PROCHECK [47].

### Analytical Ultracentrifugation (AUC)

*Ro*CBM21 and CP90 proteins at concentrations of around 1.0 mg/ml (500 µl) were used for AUC analysis. The protein and the amylose EX-I at pH of 2.5, 3, 4, 5, 6, 7, 8, 9, 10 and in different proportion of protein:amylose EX-I (1∶1, 1∶5, and 1∶10) were screened. The sedimentation coefficients (S) of the enzyme were estimated by a Beckman-Coulter XL-A optima analytical ultracentrifuge equipped with an absorbance optics unit (280 nm) and a Ti-60a titanium rotor. Sedimentation velocity analysis was performed at 40,000 rpm at 25°C with 12 nm Epon charcoal filled centerpieces. The UV absorption of the cells was scanned every 5 min for 8 h. The data from sedimentation velocity was analyzed using the SEDFIT85 [48] program and the molecular masss and sedimentation coefficients were plotted using the software Origin version 6.

### Isothermal Titration Calorimetry (ITC)

ITC measurements were made at 25°C using a Micro VP-ITC microcalorimeter (MicroCal Inc., Northampton,MA). Protein solutions (50 mM sodium acetate, pH 5.5) were first extensively dialyzed against the same buffer, and the ligands were dissolved in the same buffer. For the titrations, up to ∼30 successive 3-µl aliquots of 5 mM amylose EX-I was sequentially injected at ∼200-s intervals into a 0.04 mM protein sample and stirred at 310 r.p.m. in a 1.4331 ml reaction cell. The data were corrected for the associated heats of dilution of protein and ligand. Integrated titration curves were fit by non-linear regression using a sequential binding site model (MicroCal ORIGIN v7.0) to obtain values for *K*_a_ and ΔH°. The equation, *–RT* ln K_a_ = ΔG = ΔH − TΔS, was used to derive the other thermodynamic parameters.

### Data Analysis

Structural representations and models were generated using PyMol (Schrödinger).The RMSD on superposition of CP90 over *Ro*CBM21 was determined using iSARST [49]. The solvent accessibility of the proteins was predicted using SABLE II server (Accurate sequence-based prediction of relative Solvent AccessiBiLitiE) [44]. Dimplot [43] was used in order to get the interface residues of CP90 dimer. The intermolecular hydrogen bonds were analyzed using Discovery Studio v2.0 (San Diego: Accelrys Software Inc.). Data analysis in biophysical characterization of CP90 was performed using KaleidaGraph version 3.5b5 (Synergy Software).

### Accession Code

The atomic coordinate and structure factor have been deposited in the Protein Data Bank under accession code 4EIB for the CP90 apo structure.

## Supporting Information

Figure S1 **pH titration of of** ***Ro*** **CBM21(circle, red) and CP90 (triangle, green).** Normalized CD signals at 215 nm are displayed as a function of increasing pH from 3 to 10.
(TIF)

Figure S2 **Chemical denaturations of** ***Ro*** **CBM21(square, blue) and CP90 (circle, red).** Normalized CD signals at 215 nm are displayed as a function of increasing Gdn.HCl at pH 5.5. The curves were fitted with the nonlinear least-squares analysis according to a two-state model to show the fraction of unfolded.
(TIF)

Figure S3 **The stereo chemical spatial arrangement of amino acid residues are shown in Ramachandran plot.** The plot statistics are shown in the main text (Table 4). The Plot statistics are: residues in most favoured regions [A, B, L] −162 (83.5%); residues in additional allowed regions [a,b,l.p] −29 (14.9%) (area represented in red); residues in generously allowed regions [∼a,∼b,∼l,∼p] 1 (0.5%) (area represented in yellow); residues in disallowed regions 2 (1%) (area represented in white); number of non-glycine and non-proline residues-155(100%); number of end residues (excl. Gly and Pro)- 133; number of glycine residues (shown as triangles)- 16; number of proline residues- 4; total number of residues- 347.
(TIF)

Figure S4 **The surface elctrostatic potential of the RoCBM21 (A) and CP90 (B) displayed using the program PyMOL., with the negative potentials (red) and positive potentials (blue).** The location of major binding site residue Y32 is labelled. The evident change in the electrostatic potential near the binding site is marked in a black circle.
(TIFF)

Figure S5 **The solvent accessibilities predicted using SABLE server.** A map of increasing order of color from black to white signifying fully buried to fully exposed amino acids are represented.
(TIF)

Table S1 **Physical properties of** ***Ro*** **CBM21 and CP90.**
(DOCX)

Table S2 **Hydrogen bond contacts.** The amino acids are represented as three letter codes with the residue number and the atoms that made direct hydrogen bond contacts.
(DOCX)
