# Supplementary figures and images for: Crystal Structure of Circular Permuted RoCBM21 (CP90): Dimerisation and Proximity of Binding Sites
Source: PLoS One. 2012 Nov 30;7(11):e50488. doi: 10.1371/journal.pone.0050488 (PMC3511584; doi:10.1371/journal.pone.0050488)

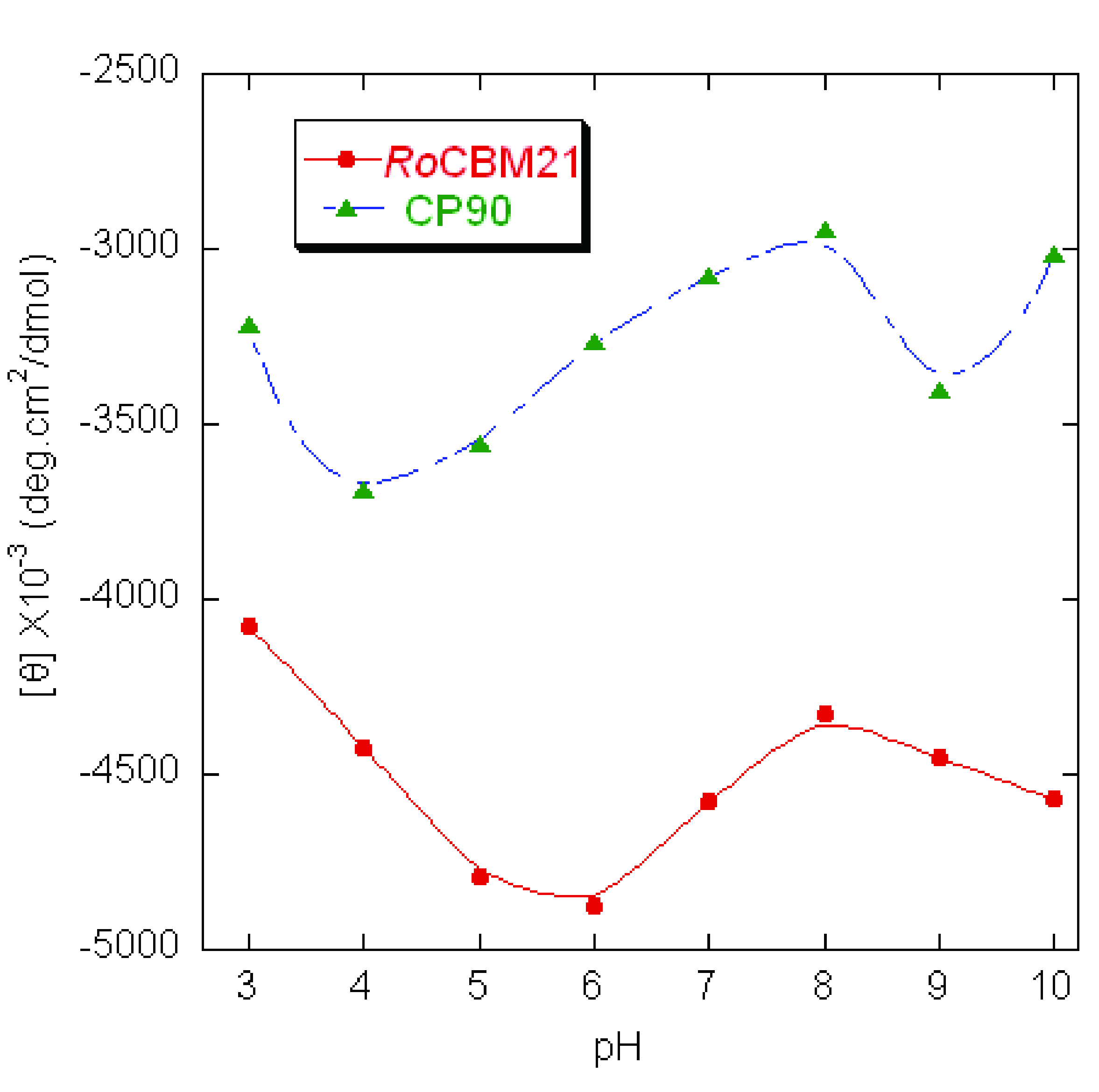

Supplement: Figure S1 — pH titration of of Ro CBM21(circle, red) and CP90 (triangle, green). Normalized CD signals at 215 nm are displayed as a function of increasing pH from 3 to 10. (TIF) [file pone.0050488.s001.tif]

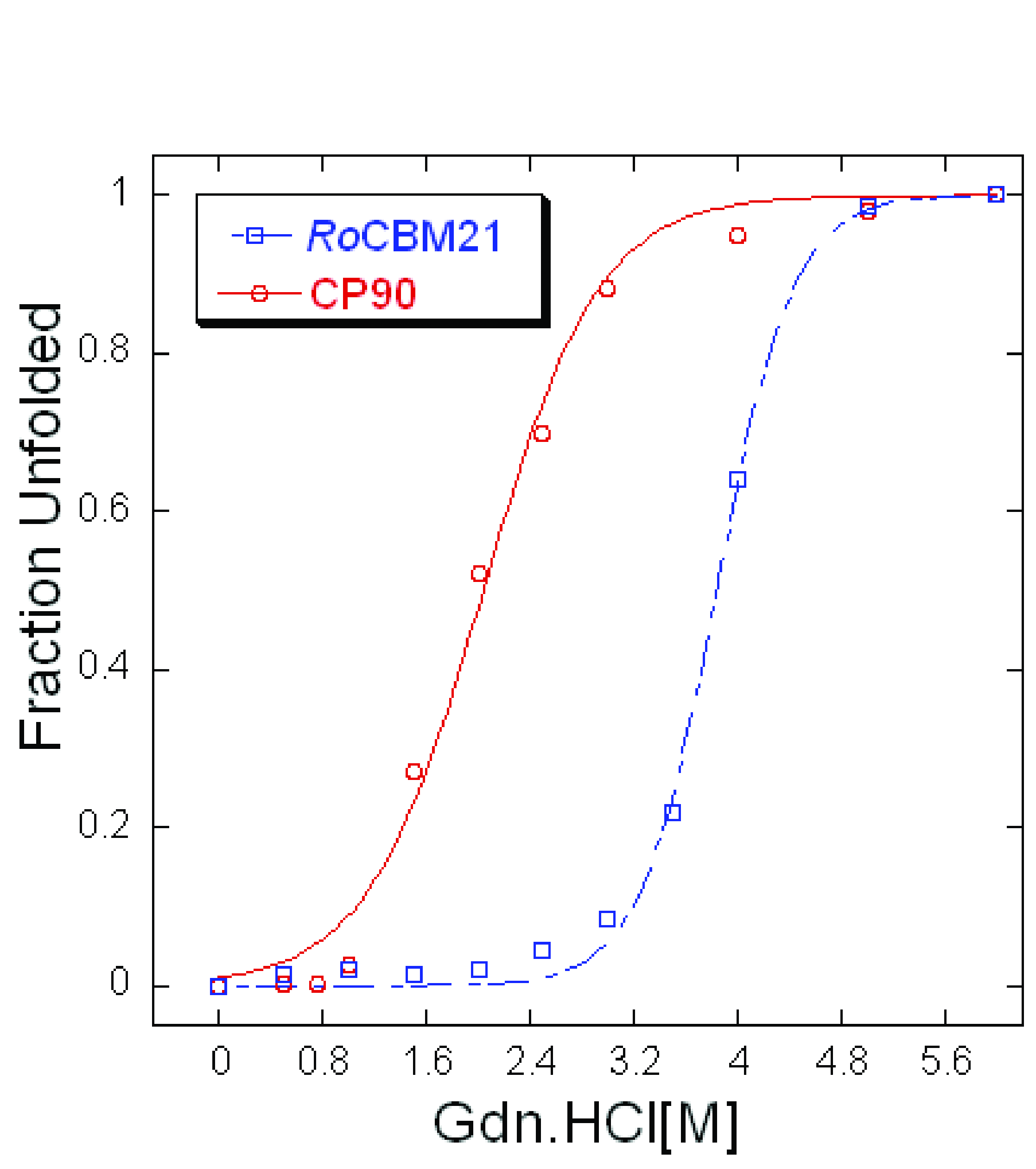

Supplement: Figure S2 — Chemical denaturations of Ro CBM21(square, blue) and CP90 (circle, red). Normalized CD signals at 215 nm are displayed as a function of increasing Gdn.HCl at pH 5.5. The curves were fitted with the nonlinear least-squares analysis according to a two-state model to show the fraction of unfolded. (TIF) [file pone.0050488.s002.tif]

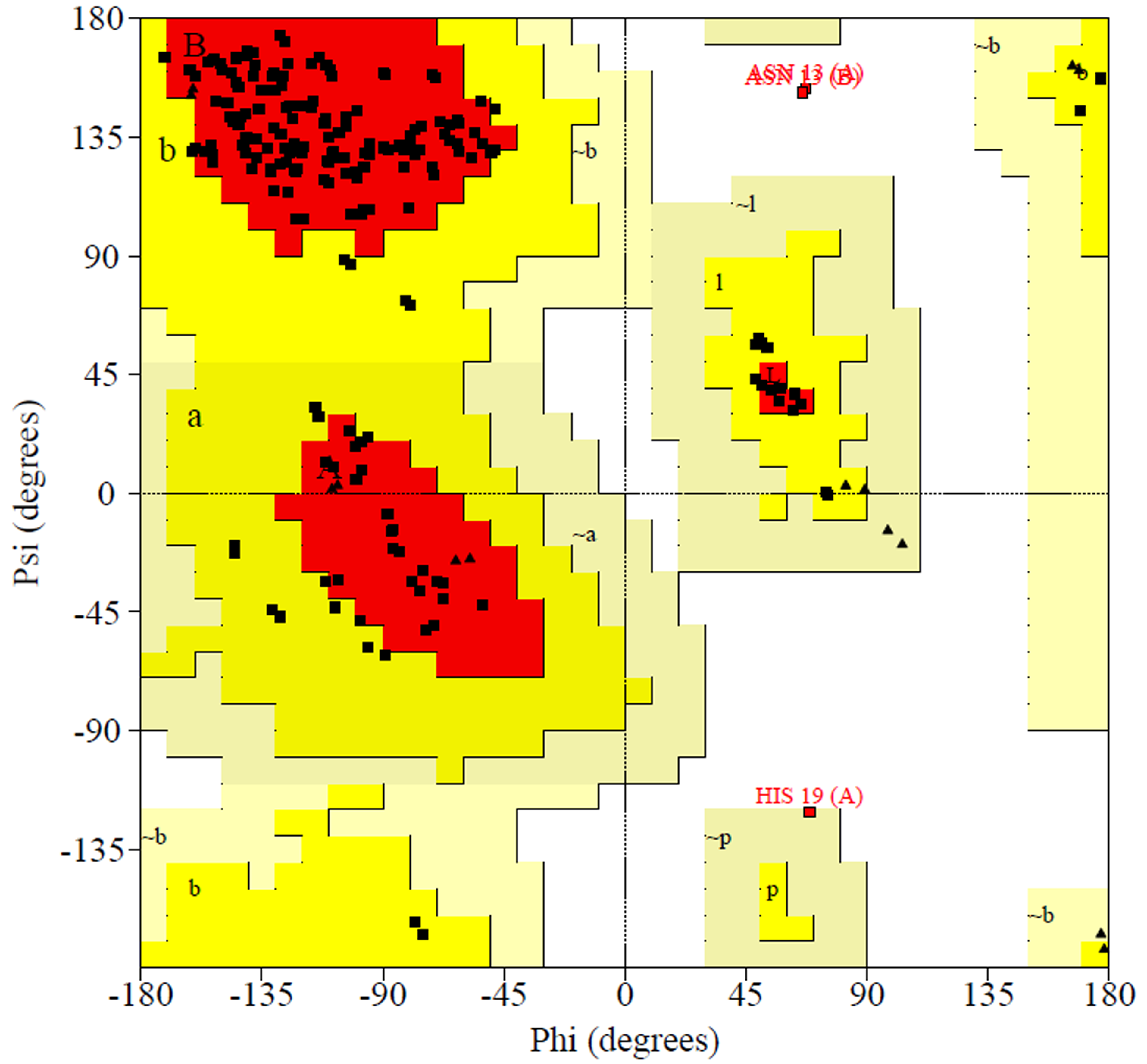

Supplement: Figure S3 — The stereo chemical spatial arrangement of amino acid residues are shown in Ramachandran plot. The plot statistics are shown in the main text (Table 4). The Plot statistics are: residues in most favoured regions [A, B, L] −162 (83.5%); residues in additional allowed regions [a,b,l.p] −29 (14.9%) (area represented in red); residues in generously allowed regions [∼a,∼b,∼l,∼p] 1 (0.5%) (area represented in yellow); residues in disallowed regions 2 (1%) (area represented in white); number of non-glycine and non-proline residues-155(100%); number of end residues (excl. Gly and Pro)- 133; number of glycine residues (shown as triangles)- 16; number of proline residues- 4; total number of residues- 347. (TIF) [file pone.0050488.s003.tif]

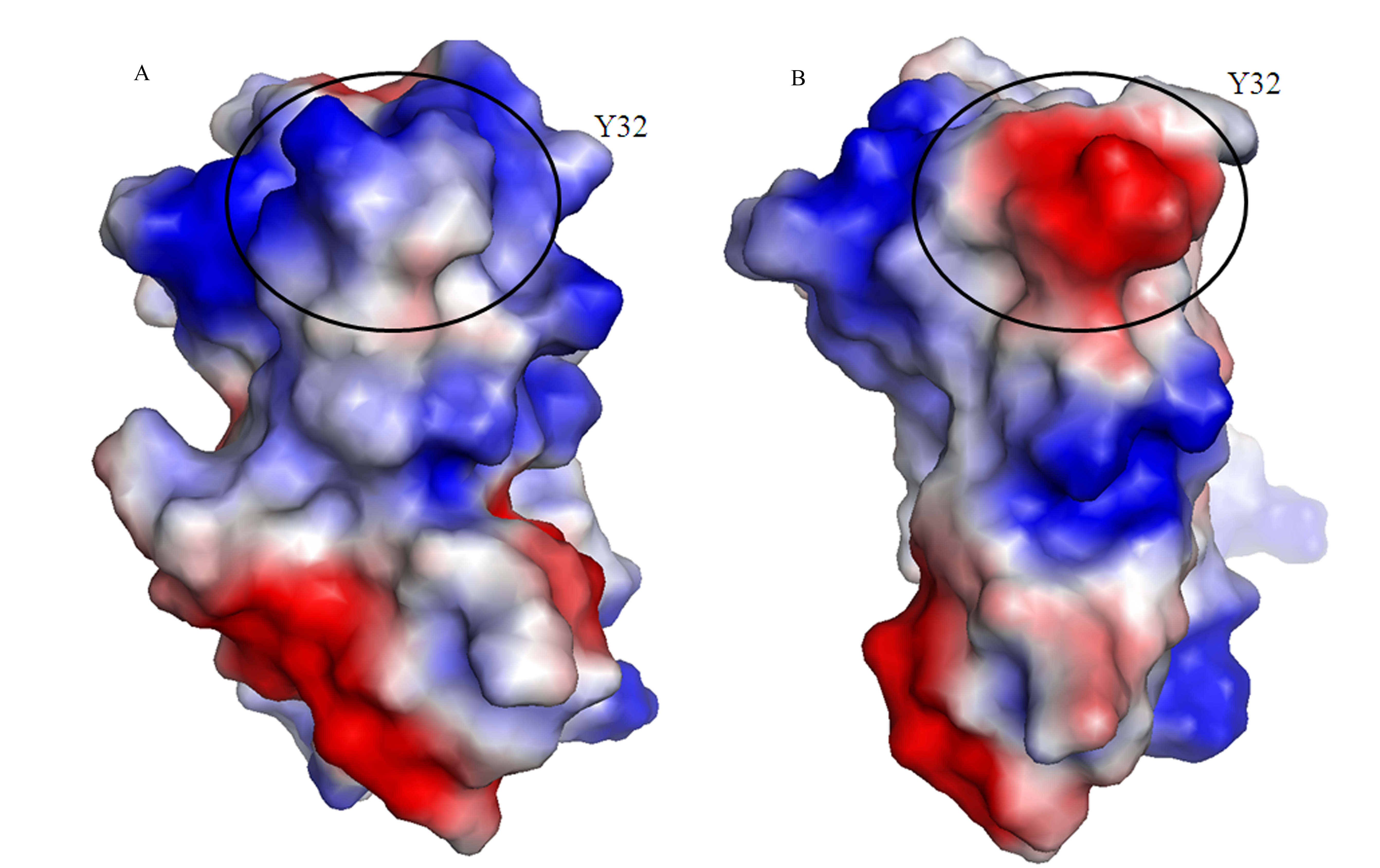

Supplement: Figure S4 — The surface elctrostatic potential of the RoCBM21 (A) and CP90 (B) displayed using the program PyMOL., with the negative potentials (red) and positive potentials (blue). The location of major binding site residue Y32 is labelled. The evident change in the electrostatic potential near the binding site is marked in a black circle. (TIFF) [file pone.0050488.s004.tifF]

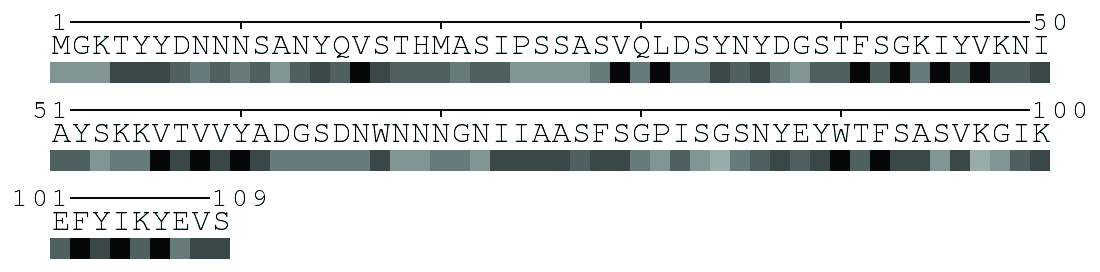

Supplement: Figure S5 — The solvent accessibilities predicted using SABLE server. A map of increasing order of color from black to white signifying fully buried to fully exposed amino acids are represented. (TIF) [file pone.0050488.s005.tif]
