# Supplementary material for: Crystal Structure of Circular Permuted RoCBM21 (CP90): Dimerisation and Proximity of Binding Sites
Source: PLoS One. 2012 Nov 30;7(11):e50488. doi: 10.1371/journal.pone.0050488 (PMC3511584; doi:10.1371/journal.pone.0050488)
Supplement: Table S1 — Physical properties of Ro CBM21 and CP90. (DOCX) [file pone.0050488.s006.docx]

|  | | |
| --- | --- | --- |
| Parameter | *Ro*CBM21 | CP90 |
| molecular mass (Da) | 11785.85 | 12054.18 |
| no. of amino acids | 107 | 109 |
| p*I* | 6.10 | 6.53 |
| Thermodynamic properties |  | |
| (i) *C_m_* (M) | 3.80 | 2.00 |
| (ii) ΔG° (H_2_O) (kJ·mol^-1^)* | 32.26 | 11.32 |
| (iii) *m* (kJ·mol^-1^·M^-1^)* | 8.40 | 5.56 |

*ΔG° = ΔG° (H_2_O) + m [denaturant] [[1](#_ENREF_1)], where *m* is a measure of the cooperativity of the unfolding

process and is proportional to the surface area exposed to solvent upon unfolding.

Supporting Reference

1. Pace CN (1986) Determination and analysis of urea and guanidine hydrochloride denaturation curves. Methods Enzymol 131: 266-280.
