# Supplementary material for: Crystal Structure of Circular Permuted RoCBM21 (CP90): Dimerisation and Proximity of Binding Sites
Source: PLoS One. 2012 Nov 30;7(11):e50488. doi: 10.1371/journal.pone.0050488 (PMC3511584; doi:10.1371/journal.pone.0050488)
Supplement: Table S2 — Hydrogen bond contacts. The amino acids are represented as three letter codes with the residue number and the atoms that made direct hydrogen bond contacts. (DOCX) [file pone.0050488.s007.docx]

| **Hydrogen bond between CP90 subunits** | | | |
| --- | --- | --- | --- |
| Subunit A | Subunit B | | Distance Å |
| Phe^78^NH | Ser^95^O^γ^ | | 2.94 |
| Ser^77^O^γ^ | Ser^95^NH | | 2.63 |
| Ala^75^NH | Ile^73^O^γ^ | | 4.15 |
| Ile^73^O^γ^ | Ala^75^NH | | 4.14 |
| Ser^95^NH | Ser^77^O^γ^ | | 3.31 |
| Ser^95^O^γ^ | Phe^78^NH | | 3.08 |
| Lys^97^NH | Lys^54^O^γ^ | | 2.93 |
|  | | | |
| **Water-mediated hydrogen bond** | | | |
| Subunit A | | Subunit B | |
| Ser^39^O^γ^ | | Thr^40^O^γ^ | |
| Lys^54^N^ζ^ | | Val^56^NH | |
| Val^56^NH | | Tyr^60^OH | |
| Thr^57^O^γ^ | | Asn^66^N^δ^ | |
| Ser^64^O^γ^ | | Asn^68^N^δ^ | |
| Asn^66^N^δ^ | | Asn^72^N^δ^ | |
| Asn^72^N^δ^ | | Asn^72^NH | |
| Ser^93^NH | | Lys^97^NH | |
| Lys^97^NH | |  | |
| Lys^97^N^ζ^ | |  | |
